# O2-4 From research to practice - BASIS Balance Strength and Safety - the Norwegian E-learning program on fall prevention

**DOI:** 10.1093/eurpub/ckac094.012

**Published:** 2022-08-29

**Authors:** Olov Belander

**Affiliations:** Department of Environmental Health, Norwegian Directorate of Health, Oslo, Norway

**Keywords:** e-learning, elderly, fall prevention, balance, strength

## Abstract

The World Health Organization and the Norwegian Directorate of Health recommends that older adults over 65 years with poor mobility should perform physical activity to enhance balance and prevent falls on 3 or more days per week. Norway has one of the highest hip fracture incidence rates in the world. Falls and hip fractures among elderly is a public health problem.

For around 10 years we have the knowledge that systematic exercise and well-designed exercise programs can reduce the rate of falls among older people living at home. The aim with this work was to develop an e-learning program to increase the knowledge about strength and balance exercises among people working at fitness centers, senior centers and nursing homes. The developing process of the e-learning program was a collaboration between the Norwegian Directorate of Health, the Norwegian University of Science and Technology and experts on e-learning. Three resource groups were also involved. The e-learning was developed from January until November 2019.

The e-learning programs BASIS is for free and available at www.basis-fallforebygging.no. Over 1200 people completed the course within the first months after release. BASIS comes in three versions and contains six modules. After completing the course, you get access to a hundred warming up, strength, balance and cool down exercises. The exercises are graded based on the functional capacity of the elderly. You can design your own programs or choose between fixed programs. The e-learning was announced and updated in 2020. Experiences will be presented.

BASIS can be an effective way to spread knowledge about exercise as well as fall prevention. The e-learning can be one important piece in the broader work to increase knowledge, change practice, prevent falls and increase the physical function in elderly people. While some organizations are changing practice fast, in many organizations changing practice seems to take longer time. The e-learning must be followed up with a change of culture, routines and practices at fitness centers, senior centers and nursing homes.

BASIS can increase knowledge about how to facilitate exercise to increase physical function in elderly at fitness centers, senior centers and nursing homes.

